# Estimating the Abundance of Widely Distributed Primates

**DOI:** 10.1002/ajp.70082

**Published:** 2025-10-26

**Authors:** Ray Hilborn, Milani Chaloupka

**Affiliations:** ^1^ School of Aquatic and Fishery Sciences University of Washington Seattle Washington USA; ^2^ Ecological Modelling Services Pty Ltd and Marine Spatial Ecology Lab University of Queensland Brisbane Queensland Australia

**Keywords:** abundance estimation, long‐tailed macaques, two‐step sampling

## Abstract

Monitoring the abundance of widely distributed animals poses many logistic challenges, and is rarely done because the wide distribution generally suggests a lack of conservation concern and thus funding. However, as there are increasing concerns about the conservation status of some widely distributed primates, evidence based management requires estimates of abundance. In this paper we review how such estimates can be done and have been done for some animals. We also explore in depth the one attempt to do so for a primate, the long‐tailed macaque. We identify weaknesses in the work that has been done and suggest how a reliable estimate could be obtained.

## Graphical abstract

This graph illustrates a key step in the process of moving from estimating habitat suitability to absolute density.

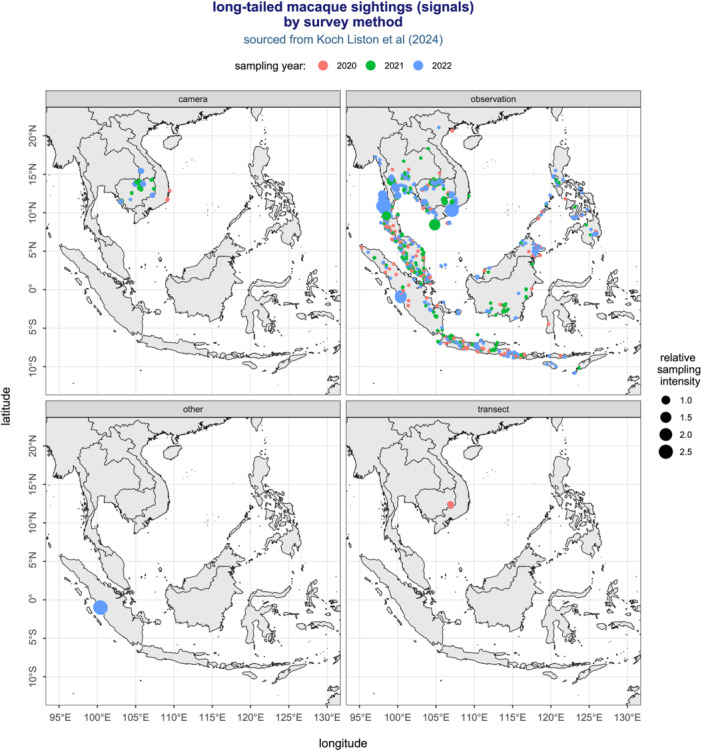

## 1 Introduction

Monitoring spatial and temporal trends in abundance of a meta‐population across a species geographic range is a necessary condition to support evidence‐informed wildlife conservation management (Lindenmayer et al. 2022; Buckland et al. 2023; Gonsalves et al. 2024). But estimating population size for a wildlife species that is dispersed over a wide geographic range is a major logistic and survey sampling challenge. Pollock et al. (2002) prescribed a cost‐effective and practical double‐sampling procedure for wildlife population monitoring based on species sighting data at landscape scale to support evidence‐informed conservation management.

That procedure comprises the following two steps: (1) extensive but inexpensive community‐sourced presence‐only sampling data to derive relative abundance indices across the entire species geographic range that are then coupled with step (2) intensive but more costly small‐scale detection/non‐detection sampling studies that are a spatial and temporal representative subset of the same presence‐only sampling sites to account for the imperfect detection inherent in Step 1. Adjusting the relative abundance indices from Step 1 with the estimated detection probabilities from Step 2 allows for the derivation of robust time series estimates of absolute species abundance at the broad landscape scale. Pollock et al. (2002, line 109) contend that this double‐sampling approach “… is the only scientifically rigorous approach” for cost‐effective wildlife monitoring at the landscape‐scale or across the entire species geographic range.

The purpose of Step 1 is to relate a series of covariates to the probability an animal was reported and thus map the relative abundance of the species across different habitat classifications based on the covariates. A range of methodologies to derive the relative abundance measures that are the goal of Step 1, some of which are reviewed in (Gelfand and Shirota 2019) and (Golding et al. 2018). Importantly, this component requires that data be collected across the entire range of environmental covariates. Thus, for example, if there were no samples at high elevations it would not be possible to estimate the relative abundance at high elevations. Although the required data are only positive observations (locations where observations were made but no sighting recorded are not required), there must have been potential observations in all habitats.

The second step generates the relationship between the relative abundance and the actual abundance. Using information on relative probability of seeing the species in different environments in Step 1, actual abundance is estimated in Step 2.

Multiple methods can be used to obtain densities including enumeration of uniquely identified individuals, mark‐recapture, line transect, distance transects, and camera traps. For primates one of the least expensive and widely used is camera trapping for estimating the primate species prevalence and occupancy at local landscape scales (Burton et al. 2015) but not for density estimation. In some camera‐trap‐based studies, individual animals can be identified to estimate abundance but this still requires using mark recapture methods and estimates of the effective area sampled (Gardner et al. 2010). But when individual identification is not possible, several methods based on unmarked animals have been proposed to estimate population metrics (Hostetler and Chandler 2015) rather than simply occupancy. However, these methods are considered unreliable for camera‐trapped based studies (Amburgey et al. 2021) and are in fact considered no better than just simply using a Poisson‐likelihood based regression to model relative abundance (Barker et al. 2018).

Twining et al. (2024) deployed a similar double‐sampling approach using extensive landscape‐scale camera trap surveys coupled with community‐sourced species sightings to derive the spatial and temporal distribution and abundance of three difficult‐to‐monitor mammal species of conservation concern (coyote *Canis latrans*, black bear *Ursus americanus*, bobcat *Lynx rufus*) across a broad range of habitats in northeastern USA. It is noteworthy here that the sampling procedure focus in (Twining et al. 2024) was on extensive camera trap survey data augmented by some community‐sourced sightings so the sampling emphasis was on Step 2 of the (Pollock et al. 2002) double‐sampling approach.

### 1.1 A Case Study in Estimating Total Abundance for a Widely Distributed Species: The Long‐Tailed Macaque (LTM)

LTMs (*Macaca fascicularis*) are distributed across SE Asia, Indonesia to Myanmar to the Philippines. They are widely used in medical testing, are often killed or sterilized due to human wildlife conflict (Moore et al. 2023) and are listed by IUCN as an invasive species (Lowe et al. 2000). However, they have also been of conservation concern (Normile 2023; Gamalo et al. 2024), listed by IUCN initially as least concern, then in 2008 as threatened then in 2022 as endangered. Thus understanding the abundance of the species is quite important for determining its conservation status but as well other reasons including national management programs. Estimating the trend in LTM abundance illustrates the challenges in estimating the abundance trends in a widely distributed primates.

The LTM has been mainly monitored at the local habitat or small‐scale rather than across the entire species range (Hansen et al. 2019; Nuttall et al. 2022; Cheyne et al. 2024). Most of those LTM surveys were based on opportunistic sampling over both space and time (Moore et al. 2023). This results in presence‐only species distribution records common in museum collections and crowd‐sourced wildlife surveys that are often referred to as citizen‐ or community‐based science (Brown and Williams 2019; Callaghan et al. 2019).

Koch Liston et al. (2024) is the only attempt to estimate the total abundance of a widespread primate, and followed the two step approach of Pollock. For Step 1 they applied a correlative species distribution modelling (cSDM) approach using the machine learning algorithm MaxEnt (Phillips et al. 2017). This was fitted to the LTM presence‐only sightings derived from the iNaturalist crowd‐sourced data repository (Callaghan et al. 2019). They derived site‐specific estimates of relative habitat suitability, and subsequently relative abundance indices, using those crowd‐sourced LTM sightings recorded opportunistically from easily accessible and hence oversampled locations in nine Southeast Asian countries. Sicacha‐Parada et al. (2021) provide an insightful discussion of the problems associated with the oversampling inherent in unstructured community‐based survey data such as the iNaturalist crowd‐sourced data repository used by (Koch Liston et al. 2024).

Importantly, the sampling procedure focus in (Koch Liston et al. 2024) was the equivalent of presence‐absence component of the (Pollock et al. 2002) double‐sampling approach outlined above, unlike the more detection/non‐detection focused landscape‐scale study in (Twining et al. 2024). Moreover, the strength of the Pollock double‐sampling procedure is that detection estimation component samples in depends on being a spatial and temporal representative subset of the same community‐sourced presence‐only sampling sites—but that was not generally the case in the sampling procedure deployed by (Koch Liston et al. 2024) to estimate the distribution and abundance of the LTM across the entire species range, which is a vast area ca 4.2 M km^2^ and encompasses ca 650 million people.

We fully appreciate the challenges with sampling the LTM across the species range and acknowledge the efforts undertaken recently by (Koch Liston et al. 2024) to address these challenges. However, we have a number of concerns about the sampling and modeling procedures used in that recent assessment that we address below with our explicit intent being to highlight the limitations of current efforts and propose some approaches to improve future surveys to estimate the abundance of widely distributed primates.

Below, we revisit the history and challenges of estimating the total abundance of LTMs and offer our suggestions as to what additional data would be needed to acquire a more robust and realistic estimate. We will use the example of (Koch Liston et al. 2024) to evaluate key issues in such ambitious attempts and are guided by the double‐sampling procedure proposed by (Pollock et al. 2002) outlined above and the more robust statistical modeling of presence‐only data using recent developments in spatial Poisson point process models (Gelfand and Shirota 2019; Piironen et al. 2022) to estimate relative abundance trends that we discuss in more detail below.

The highest densities of LTMs are found where they are intentionally provisioned such as temples or adjacent to agriculture and settlements where they have access to crop raiding and garbage. In forests, higher densities are found in mangroves, riverine forests, secondary forests, with lower densities in primary forests, and even lower densities in higher altitude forests (Fooden 1995). Below, we summarize local estimates of LTM densities at sites from across their geographic range (Table 1) and then review attempts to characterize abundance across that large range.

**Table 1:** Published density estimates of LTMs in different habitats.

Country	Source	Year	Habitat	Density #/km^2^	Method
Indonesia	Crockett (1980)	1973	Swamp mangrove	80.9	Strip transect
Indonesia	Crockett (1980)	1973	Swamp Mixed mangrove	143.0	Strip transect
Indonesia	Crockett (1980)	1973	Swamp riverbank	61.2	Strip transect
Indonesia	Crockett (1980)	1973	Swamp selective logging	29.9	Strip transect
Indonesia	Crockett (1980)	1973	Swamp secondary forest riverbank	25.5	Strip transect
Indonesia	Crockett (1980)	1973	Lowland Primary Forest	46.1	Strip transect
Indonesia	Crockett (1980)	1973	Lowland primary forest riverbank	37.6	Strip transect
Indonesia	Crockett (1980)	1973	Lowland secondary forest	74.4	Strip transect
Indonesia	Crockett (1980)	1973	Lowland secondary forest riverbank	28.6	Strip transect
Indonesia	Crockett (1980)	1973	Lowland secondary forest rubber grove	72.5	Strip transect
Indonesia	Crockett (1980)	1973	Lowland secondary forest scrub grassland	33.1	Strip transect
Indonesia	Crockett (1980)	1973	Lowland secondary forest riverbank	112.0	Strip transect
Indonesia	Crockett (1980)	1973	Hill primary forest	97.8	Strip transect
Indonesia	Crockett (1980)	1973	Hill secondary forest	135.6	Strip transect
Indonesia	Crockett (1980)	1973	Hill secondary forest rubber grove	51.0	Strip transect
Indonesia	Crockett (1980)	1973	Hill secondary forest scrub, grassland	11.2	Strip transect
Indonesia	Crockett (1980)	1973	Submontain Primary forest	0.0	Strip transect
Indonesia	Crockett (1980)	1973	Submontain Secondary forest	0.0	Strip transect
Indonesia	Perwitasari‐Farajallah et al. (2001)	1991	Whole island	166.7	Unknown
Indonesia	Supriatna (1996)	1993	Peat swamp forest	88.5	Strip transect
Indonesia	Supriatna (1996)	1993	Tidal Forests	78.0	Strip transect
Indonesia	Supriatna (1996)	1993	Hill sites	10.4	Strip transect
Indonesia	Supriatna (1996)	1993	Lowland forests	31.9	Strip transect
SE Asia	Fooden (1995)	1995	Provisioned	100.0	Unknown
SE Asia	Fooden (1995)	1995	not provisioned	50.0	Unknown
Viet‐Nam	Son (2004)	2000	Provisioned mangrove	62.0	Individual observations
Indonesia	Leeson et al. (2004)	2001	Lowland Forest	448.0	Strip transect
Singapore	Sha et al. (2009)	2007	Urban forest	60.1	Individual observations
Indonesia	Brotcorne (2009)	2009	Provisioned and crop raiding	1,660	Individual observations
Indonesia	Yanuar et al. (2009)	2009	Lowland Forest	10.7	Distance transect
Indonesia	Yanuar et al. (2009)	2009	Hill	6.6	Distance transect
Indonesia	Afendi et al. (2011)	2010	Forest	11.46	Unknown
Indonesia	Fauzi et al. (2020)	2011	Forest	5	Strip transect
Singapore	Riley et al. (2015)	2011	Urban forest	6.9	Individual observations
Indonesia	Anggraeni et al. (2013)	2012	Forest	55	Individual observations
Indonesia	Brotcorne et al. (2014)	2011	Forests	70	Individual observations
Thailand	Schurer et al. (2019)	2017	Provisioned temple	3,650	Individual observations
Indonesia	Hansen et al. (2019)	2018	National park	41.4	Distance transect
Cambodia	Agger (2023)	2020	Park	0.5	Distance transect
Viet‐Nam	Koch Liston et al. (2024)	2020	Park	0.7	Unknown

The conservation status of LTMs around their range is highly variable. They appear to be extinct in Bangladesh, and are reported to be almost absent in Laos (Hansen et al. 2022) and at quite low densities in some parks in Cambodia and Viet Nam. Recent studies show relatively high densities in parts of Indonesia, and they are seen at very high densities near human settlements and agriculture (see Table 1). Their density depends not only on habitat but removals either for human food, sale, or pest control. In Malaysia roughly 50,000 individuals are reported to be removed from the wild each year as due to conflict with humans (Hilborn and Smith 2023). Where they are now rare, the most common explanation is removals (Hansen et al. 2022). To use the method of (Pollock et al. 2002) to estimate their overall abundance with any reliability, density estimates cannot be from a few locations, but from locations covering their both different habitats but different past and present levels of removals.

The first published estimate of total abundance of LTM is 5 million individuals from (Fooden 1995, p. 54), “Based on rough estimates of population density and remaining available habitat, K. S. MacKinnon (1986, p. 111) calculated provisionally that the population of *M. fascicularis* in Indonesia at that time was ca. 3,726,860, and J. R. MacKinnon and MacKinnon (1987, p. 189) calculated that the population in mainland Southeast Asia north of West Malaysia was 309,360. If these provisional calculations are reasonable, the total population of this species about 10 years ago in its entire natural range‐which, in addition to Indonesia and mainland Southeast Asia north of West Malaysia, includes the Nicobar Islands, Malaysia, Brunei, and the Philippines—may have been approximately 5 million.”

The MacKinnon (1986) estimate of 3,726,860 LTMs in Indonesia that Fooden (1995) later used was derived by estimating that there were 73,371 km^2^ of remaining primary habitat in Indonesia with a mean density of 30 individuals/km^2^ and 38,750 km^2^ of remaining secondary habitat with a mean density of 40 individuals/km^2^. The area of each habitat was cited on page 99, as “National Conservation Plan for Indonesia, FAO‐INs/78/061.” No source is given for the density estimates but the author notes on page 107 “[t]he conservative working density estimates for each species (Table 8.3), subjective as they are, are meant to give a “safe” figure applicable to the whole area of habitat considered.”

The 309,360 estimate for Indochina in MacKinnon and MacKinnon (1987) came from an estimated 15,468 km^2^ of remaining coastal and riverine forest in Indochina with an average density of 20 individuals/km^2^. There is no documentation, however, of how the estimates for areas other than Indonesia and Indochina were derived but it must have been roughly 1 million individuals to bring the total to 5 million. No measures of uncertainty are available and given the rather simple methods this estimate must be considered very uncertain.

Therefore, it is difficult to estimate the reliability of the total estimated presented in (Fooden 1995) and it would provide a very unreliable starting point for any attempt to estimate a rate of decline.

The second estimated total abundance cited by Hansen et al. (2022) and Gamalo et al. (2024) is 3 million by (Fooden 2006). However, there is no statement in that paper of how that number was derived and it cites other sources that never mention the number. Thus it is difficult to assess the credibility of the 3 million estimate.

As noted above, Koch Liston et al. (2024) is the first significant attempt since (Fooden 1995) to estimate the global abundance of LTMs. Their approach generally follows that suggested by (Pollock et al. 2002). To calculate relative abundance they used presence records, almost all of which come from citizen science observations uploaded to the iNaturalist platform, augmented by smaller numbers of sightings recorded by researchers. These observations are combined with 16 habitat covariates to generate model of relative abundance as a function of the covariates. Then they estimate the relationship between habitat preference and density. It is difficult to determine exactly how this was done but appears to be using camera trap data and density estimates from a single sampling location. They then integrate across the map of habitat preference, converting habitat preference to density to obtain estimates by country and globally.

**Figure 1:**
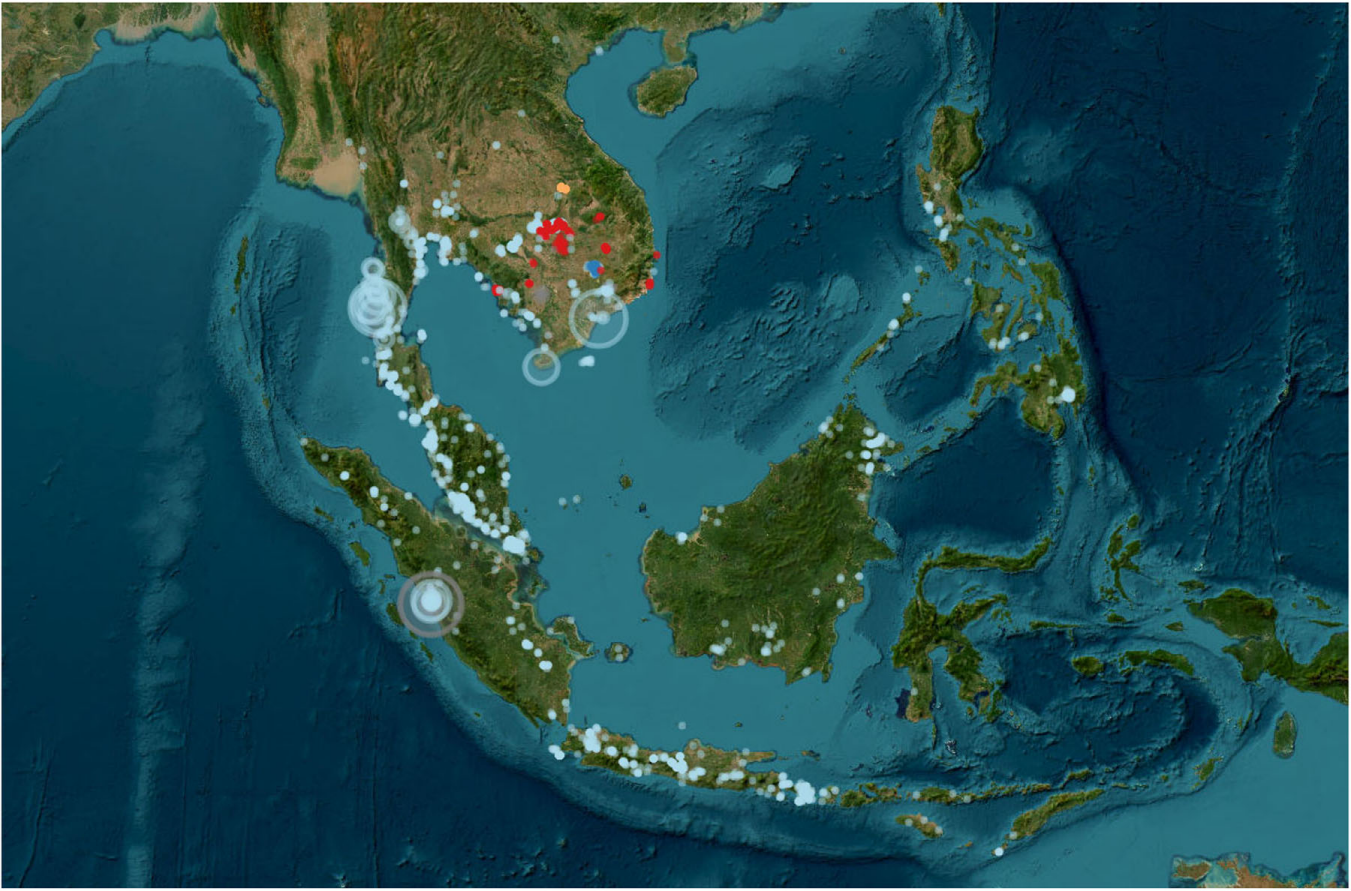
Map of recorded LTM sightings derived from various sampling methods. Red = camera traps, blue = direct sightings, orange = line transect. The size of the circle is proportional to the total number of sightings. Drawn from code supplied in supplemental materials of (Koch Liston et al. 2024).

**Figure 2:**
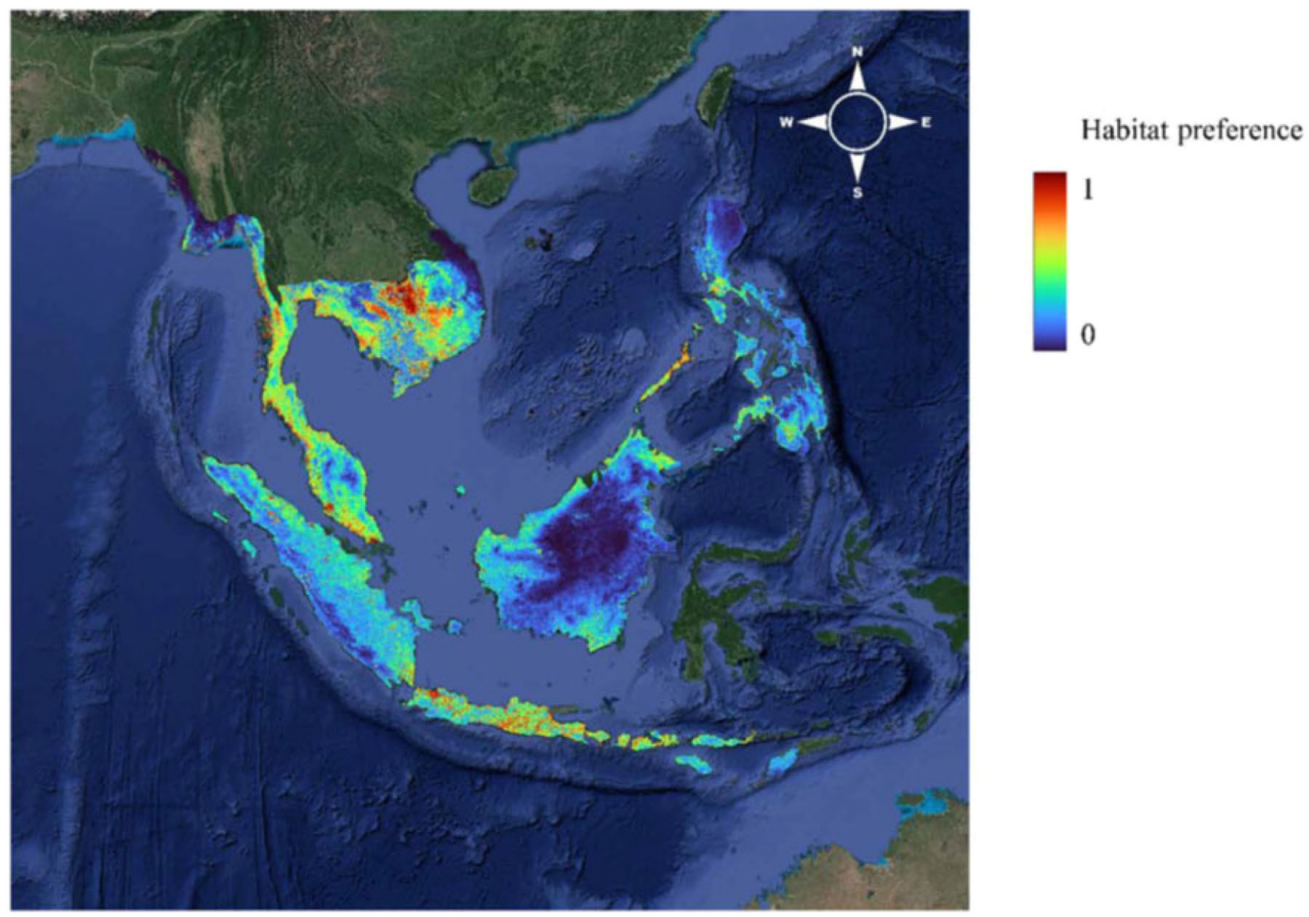
Estimate habitat preference map redrawn from (Koch Liston et al. 2024).

The estimate of habitat preference relies on sightings by each of the methods using the machine learning algorithm MaxEnt (Phillips et al. 2006; Phillips et al. 2004) that uses presence of sightings to estimate a species distribution preference. Renner et al. (2015, p. 377) note that “in order to interpret intensity as relative abundance of individuals per unit area, the intensity of presence records should be proportional to the intensity of individuals (i.e., abundance) of the species” They caution that when data come from data collection programs like iNaturalist, the intensity of sightings is primarily dependent on the density of people using iNaturalist not the density of the species of concern. This is easily seen in the distribution of sightings in Figure 1 where we see very strong concentration of sightings in heavily visited coastal Malaysia, Java and Thailand, with an almost total absence of sightings in Borneo and relative few in Sumatra. It is not surprising then that the habitat preference map (Figure 2) shows the high habitat preferences largely in the areas where many sightings were observed, and that the highest habitat preference is seen in Cambodia where camera traps were most common. As shown in Table 1, estimated densities in this region of Cambodia were the lowest ever recorded.

MaxEnt (or MaxNet) is one of the most widely used algorithms for cSDMs (Valavi et al. 2022) and is based on using species presence‐only data coupled with a large number of randomly generated background sample locations (pseudo‐absences) across the surveyed landscape. It is noteworthy that a range of concerns have been repeatedly raised about the application of MaxEnt for cSDMs with presence‐only data because (1) it is well known to provide overly complex model fits with poor spatial transferability (Vollering et al. 2019) and (2) MaxEnt fails to account explicitly for spatial variation in the presence‐only observations, which is the main point of a data‐informed cSDM (Gelfand and Shirota 2019).

Essentially all of the high habitat preference areas are in Cambodia where camera trapping was intense and coastal Thailand, Coastal Malaysia and Java where tourists are common. Less visited Sumatra, and Borneo all are low habitat preference. Thus this habitat preference map seems unlikely to actually reflect where LTM occurs but rather where there were observations available and this makes the entire analysis suspect.

**Figure 3:**
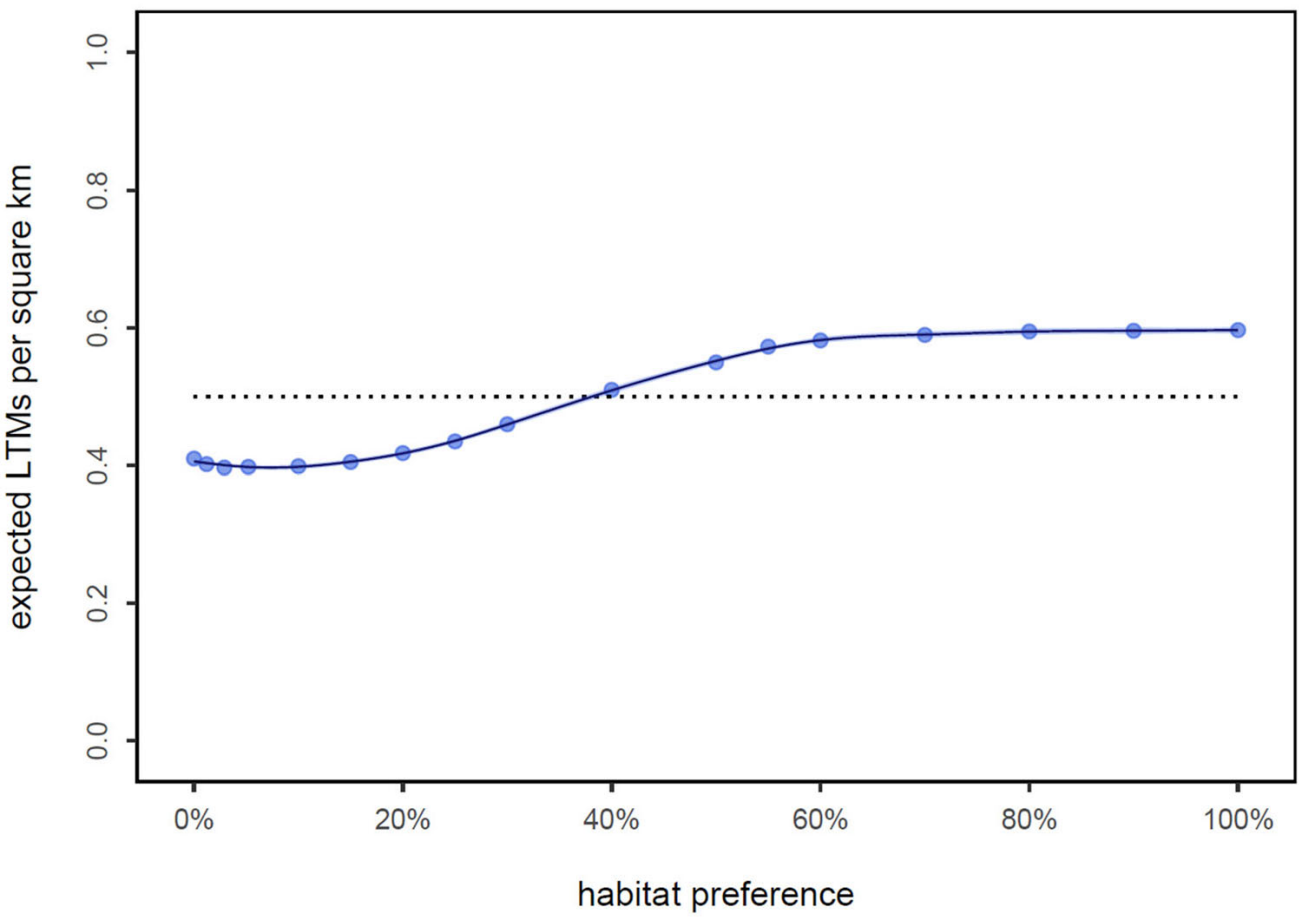
The estimated relationship between habitat preference and expected long‐tailed macaque numbers per km^2^. Redrawn from (Koch Liston et al. 2024).

The next step in their approach is estimating the relationship between habitat preference and density is shown in Figure 3. There are two surprising results from this analysis. First is the almost flat relationship between habitat preference and LTM density, going from 0.38 to 0.53 suggesting almost no relationship between “habitat preference” and LTM density. However, such a result would not be unexpected if the real determinant of sighting density is where tourists and camera traps are and that is unrelated to the 16 environmental variable that were used in their Step 1 model. These 16 variables included 6 climatic variables, 3 topographic variables, 5 land cover classes and distance to the coast and roads.

Even more surprising is the low predicted LTM densities that leads to their global estimate of 904,638 as an upper limit. As we saw in Table 1, among recent densities estimates using line transect distance methods in unprovisioned sites there were two low points in Cambodia and Viet‐Nam with densities below 1/km^2^, and other estimates ranging from 6 to 40/km^2^. Koch Liston et al. (2024) used the Vietnamese and Cambodian estimates to do consistency checks. This very low density range is remarkable since the most recent density estimates available are 41/km^2^ from Java by the corresponding author of their paper. The Koch Liston et al. (2024) model would predict not 41/km^2^, but less than 1/km^2^ in that region of Java.

Another quality check on their prediction is the overall estimate for Malaysia of 119,499 animals. The Malaysian Wildlife Department reports culling ca. 50,000–60,000 individuals per year for the last decade. This level of removals from a population of only 119,499 animals seems unlikely. If, however, Koch Liston et al. (2024) had tuned their relationship between habitat preference and density to the Hansen et al. (2019) density estimate of 41/km^2^, then their estimate of the Malaysian population size estimate would be over 2 million animals and annual removals of 60,000 would be easy to accept.

## 2 A Practical Design for Estimating LTM Abundance

Here we describe how total LTM abundance could be reliably estimated using the Pollock method for a large region, and to provide a global estimate this would need to be duplicated in all regions where LTMs are thought to be found in significant numbers. Regions would include, at a minimum, most of Indonesia, Malaysia and Thailand. For Step 1 presence data from existing iNaturalist and other observations could be employed but would not provide representative coverage across habitat types. This would need to be supplemented by teams collecting presence/absence across a range of habitats by their own observation and asking local people. This could be done by driving on roads, and stopping every 10 km, interviewing local residents and recording habitat type, human population density, elevation, any evidence for provisioning, or efforts to reduce abundance. If some habitats were not accessible by road, the team would need to walk or boat into those. Step 2 would require distance transect studies across enough sites to reliably estimate the relationship between habitat characteristics and density. The limited number of existing density estimates could be incorporated into this step.

Such a study for a large area such as the major Indonesian islands, would be expensive, likely taking a field team several months A critical question for expanding beyond a single region would be whether the relationship between density and habitat characteristics was consistent from region to region, thus for each region would a new set of distance transect studies be required or could one set of such measurements be applied to all regions. This would seem unlikely as the level of control effort may be quite different between countries or regions with a specific country.

## 3 Conclusions

We submit that, in theory it would be possible to estimate the global abundance of a primate species such as LTMs using the two‐step approach suggested by (Pollock et al. 2002) but far more data would be needed to do so reliably. The Koch Liston et al. (2024) recent attempt to do so is flawed and results in a serious underestimate of the global abundance of the species. Using presence‐only data would work for the first step, but the coverage of the data set would need to be much more representative of the conditions impacting the presence of the species than using iNaturalist opportunistic sightings records or any other spatially biased community‐based sampling program. The data set would include broad or landscape scale presence‐absence data that can be rapidly collected, coupled with local density estimates from various transect methods. In the absence of individual identification it seems unlikely that camera traps can be used to estimate absolute densities but they can provide valuable presence‐absence data.

With respect to the trend in abundance of LTMs, the Fooden (1995) estimate was derived using a very rough method that implies significant uncertainty. Koch Liston et al. (2024) estimates a totally unrealistic relationship between habitat suitability and density, one that is totally inconsistent with all but two of the existing density estimates, and those two are the lowest LTM density estimates recorded anywhere. Thus we conclude that there is no reliable estimate of the long‐term trend in LTM abundance. Hence, we contend after careful consideration of Koch Liston et al. (2024) that a more rigorous assessment of LTM meta‐population trends needs to be undertaken to support credible and evidence‐informed IUCN status determinations for this species across the entire southeastern Asian terrestrial landscape.

The needed field work to assess the global, or even regional abundance has not been done perhaps because historically they were thought to be so abundant and the cost would be considerable. If, as we suggest, the Koch Liston et al. (2024) abundance estimates are much lower than the actual abundance then it is likely LTMs are not of global conservation concern. A single regional study using the method we outlined could perhaps resolve the uncertainty. Given that Indonesia was initially estimated to be the home of the majority of LTMs and seems likely to remain so, then a single regional study in Sumatra might be a high priority.

## Ethics Statement

The authors have nothing to report.

## Data Availability

The authors have nothing to report.
